# Transforming the Management of Oligometastatic Non-Small Cell Lung Cancer in the Era of Immunotherapy and Targeted Therapy

**DOI:** 10.3390/cancers17182982

**Published:** 2025-09-12

**Authors:** Yohei Kawaguchi, Satoshi Takahashi, Yoshihisa Shimada, Yujin Kudo, Norihiko Ikeda

**Affiliations:** Department of Surgery, Tokyo Medical University Hospital, Tokyo 1600023, Japanzenkyu@za3.so-net.ne.jp (Y.S.); ykudo@tokyo-med.ac.jp (Y.K.);

**Keywords:** oligometastatic lung cancer, non-small cell lung cancer, local ablative therapy, synchronous metastasis

## Abstract

Oligometastatic non-small cell lung cancer (omNSCLC) is increasingly recognized as a clinically actionable subset, characterized by a limited number of metastatic lesions and the potential to benefit from combined systemic and local treatments. Advances in systemic therapy, particularly immune checkpoint inhibitors (ICI) and molecular targeted agents, have led to prolonged disease control in selected patients. Prospective clinical trials have shown that incorporating local therapy into systemic treatment can improve progression-free and overall survival outcomes in carefully selected patients with metastatic disease. Radiotherapy has increasingly become the preferred modality for local consolidation, especially when surgical options are limited or impractical. Key factors influencing treatment selection include biomarker status, the number and distribution of metastases, and the response to the initial systemic therapy. The optimal timing and modality of local interventions remain under investigation. This review examines the evolving treatment strategies for omNSCLC, primarily from the perspective of systemic therapies. By contextualizing local treatment modalities within the framework of immunotherapy and targeted therapy, we reinterpret current evidence to clarify when and how local therapy contributes to long-term disease control.

## Simple Summary

Oligometastatic non-small cell lung cancer is a form of lung cancer where the disease has spread, but only to a limited number of sites. This group of patients is important because they may live longer if treatment is carefully planned. Modern drug treatments, such as immunotherapy and targeted therapies, have helped some patients achieve longer control of the disease. In addition to drug treatment, local approaches such as surgery or radiotherapy can be used to directly treat cancer in the chest or metastatic sites. Radiotherapy has become the most common option when surgery is not possible. The choice of treatment depends on factors such as the number and location of metastases, the patient’s response to drug therapy, and the presence of biological markers. This review explains how local and systemic treatments can be combined to improve survival and guide future treatment strategies.

## 1. Introduction

Oligometastatic non-small cell lung cancer (omNSCLC) occupies an intermediate position on the metastatic continuum, situated between disease confined to the thorax and widely disseminated stage IV cancer. Originally described by Hellman and Weichselbaum as a potentially curable state, omNSCLC is defined as a limited number of metastatic deposits that remain amenable to definitive local therapy. In 2019, the IASLC, together with the EORTC, ESTRO, and ESMO, issued a consensus statement that operationalized this concept: synchronous oligometastasis is present when five or fewer metastatic lesions involve no more than three organs, provided each lesion can be ablated safely through surgery, stereotactic radiotherapy, or another local modality [1].

Contemporary population-based registries indicate that approximately one-third of newly diagnosed stage IV NSCLC cases meet these anatomical criteria. This subgroup attains a median overall survival of nearly two years, almost twice that of unselected metastatic cohorts receiving systemic therapy alone [2]. Reflecting the prognostic relevance of this disparity, the eighth edition of the AJCC/UICC TNM classification further stratifies metastatic disease, distinguishing limited extrathoracic spread (M1b) from diffuse multi-organ involvement (M1c). This refinement highlights the biological heterogeneity of stage IV NSCLC and provides a staging framework that justifies more aggressive multimodal strategies in carefully selected patients with a low metastatic burden [3]. Beyond the oligometastatic definition and M1b/M1c stratification, contemporary stage III consensus statements emphasize multimodality integration and immunotherapy consolidation—principles that conceptually bridge thoracic-confined disease to omNSCLC, where systemic therapy and local ablative therapy (LAT) are likewise orchestrated to maximize durable control [4]. Building on these definitions and epidemiological observations, therapeutic approaches for omNSCLC have evolved from retrospective reports to early-phase trials, suggesting improved survival with the integration of LAT. Recently, randomized studies have provided prospective evidence supporting this multimodal paradigm, although consensus on patient selection and timing remains unsettled. These considerations frame the rationale for our review, which reinterprets the current evidence in the era of immunotherapy and targeted therapy.

The management of omNSCLC now relies on two pivotal therapeutic advances.

### Potent Systemic Drugs

Immune checkpoint inhibitors (ICIs) have supplanted platinum doublets as the first-line standard for tumors lacking actionable oncogenic drivers. Pivotal phase III trials, including KEYNOTE-024, KEYNOTE-042, and Impower 110, have demonstrated that PD-1/PD-L1 antibodies administered as monotherapy significantly prolong both progression-free and overall survival compared to chemotherapy [5,6,7]. Subsequent combination studies (for example, KEYNOTE-189 and KEYNOTE-407) confirmed that the addition of pembrolizumab to platinum chemotherapy confers further survival benefit across all PD-L1 expression statuses [8,9]. Furthermore, dual checkpoint blockades, such as nivolumab plus ipilimumab (CheckMate 227 and CheckMate 9LA) or durvalumab plus tremelimumab (POSEIDON), have shown favorable survival benefits even in PD-L1-negative patients [10,11,12]. Crucially, ICIs exhibit clinically meaningful intracranial activity, extending durable systemic control to central nervous system sanctuary sites.

In oncogene-addicted diseases, highly selective tyrosine kinase inhibitors (TKIs) have fundamentally altered outcomes by inducing rapid, profound, and central nervous system-penetrant responses. For epidermal growth factor receptor (EGFR)-mutated NSCLC, the third-generation osimertinib superseded earlier agents after the FLAURA trial showed objective response rates > 70%, a median progression-free survival of 18.9 months, and a 52% reduction in the risk of CNS progression [13]. Recently, combination regimens that integrate TKIs with concurrent platinum–pemetrexed chemotherapy have begun to outperform TKI monotherapy. The phase III FLAURA2 trial showed that first-line osimertinib plus chemotherapy yielded a 38% relative reduction in the risk of progression or death compared with osimertinib alone (hazard ratio, 0.62) and produced higher objective-response and intracranial-control rates [14]. Comparable advances have been achieved for other oncogenic drivers, including rearrangements of the anaplastic lymphoma kinase (ALK) gene (for example, EML4–ALK; targeted by alectinib and lorlatinib), ROS1 fusions (entrectinib), rearrangements of the RET (selpercatinib), exon 14-skipping alterations in MET (tepotinib), and the missense mutation in KRAS-G12C (sotorasib)—broadening the targeted-therapy armamentarium [15,16,17,18,19,20].

Image-guided surgery, stereotactic body radiotherapy (SBRT), and percutaneous ablative techniques now allow the definitive eradication of limited metastatic deposits with low morbidity, achieving durable local control in carefully selected patients. Among these options, SBRT has undergone substantial refinements in planning accuracy, motion management, and dose conformity, enabling the safe delivery of ablative biological doses (>100 Gy) even to lesions that were historically considered unresectable because of their proximity to critical organs. As a result, anatomically challenging sites such as the adrenal gland, para-aortic nodes, and deep pelvic lymphatics can now be treated with one-year local-control rates exceeding 90% and grade ≥ 3 toxicity below 5% [21,22].

These advances have shifted the clinical debate from whether long-term survival is attainable to how best to integrate potent systemic therapy with LAT to improve patient outcomes. Therefore, current international consensus statements recommend a multidisciplinary algorithm in which systemic agents eradicate occult micrometastases, while LAT removes every radiographically visible lesion whenever technically and physiologically feasible [23].

This narrative review traces omNSCLC management in the era of ICIs and TKIs and highlights the role of LAT in treatment.

## 2. Data Source and Search Date

We performed a single comprehensive search of PubMed/MEDLINE on 15 May 2025. No other databases or trial registries were used in this study. Filters were set to humans, English language, and clinical trials (Phase I–III).

### 2.1. Core Search Keywords

The keywords were grouped into five conceptual domains.

1. Disease: “non-small cell lung cancer”, “non-small-cell lung carcinoma”, and “NSCLC”;

2. Metastatic state: “oligometastatic”, “oligometastasis”, “limited metastatic”, and “synchronous metastatic”;

3. Systemic therapy: “immune checkpoint inhibitors”, “pembrolizumab”, “nivolumab”, “ipilimumab”, “durvalumab”, “tremelimumab”, “atezolizumab”, “Tyrosine kinase inhibitors”, “gefitinib”, “erlotinib”, and “osimertinib”;

4. Local ablative therapy: “stereotactic”, “SBRT”, “stereotactic body radiotherapy”, “stereotactic ablative radiotherapy”, “SABR”, “stereotactic radiosurgery”, “SRS”, “radiotherapy”, “irradiation”, “surgery”, “resection”, “local ablative therapy”, and “ablation”;

5. Prospective trial: “randomized”, “phase I”, “phase II”, “phase III”, and “trial”.

Terms were combined with Boolean operators and restricted to the Title/Abstract field, and MeSH mapping was allowed for the humans filter.

### 2.2. Eligibility Criteria

Adults (≥18 years) with histologically confirmed synchronous omNSCLC.

Prospective Phase I–III trials evaluating ICIs or TKIs delivered together with curative-intent LAT (surgery, SBRT/SABR, SRS, or proton therapy).

Reporting at least one survival endpoint (progression-free survival [PFS] and/or overall survival [OS]); sample size ≥ 10.

The exclusion criteria were oligoprogression, oligorecurrence, retrospective study designs, abstracts without peer review, and non-English publications.

The initial search yielded 68 unique records, which the first author (Y.K.) screened independently at the title and abstract level using a standardized eligibility checklist. Full-text articles were retrieved for potentially relevant citations. Each document was then evaluated against the pre-specified inclusion and exclusion criteria, covering study design (prospective only), population size (≥10 patients), treatment modality (ICI + LAT or TKI + LAT), and outcome reporting (progression-free or overall survival). Duplicate publications, such as interim analyses or secondary subgroup reports, were identified by cross-referencing trial acronyms and ClinicalTrials.gov identifiers and were removed. Following this two-stage appraisal, seven prospective trials satisfied all criteria: five investigated ICI combined with LAT, and two investigated TKI combined with LAT (Table 1).

## 3. ICI and LAT

The main results of the clinical trials included in this review are shown in Table 2. Five prospective studies that met the review’s eligibility criteria collectively illustrated the therapeutic value of integrating ICI with LAT in omNSCLC. In a phase II study by Bauml et al., pembrolizumab delivered after complete LAT produced a median PFS of 19.1 months and a two-year OS rate of 77.5%, figures that clearly exceed historical benchmarks for single-agent pembrolizumab [24]. Bassetti et al. employed multisite SBRT given concurrently with dual PD-L1 and CTLA-4 inhibition; although grade ≥ 3 toxicity reached 40%, the regimen achieved a one-year PFS of 59%, underscoring the potential efficacy of intensified immunoradiotherapy when all macroscopic disease is sterilized locally [25]. The sequencing question was addressed by Bestvina et al., whose randomized phase I comparison of concurrent versus sequential nivolumab plus ipilimumab with multisite SBRT showed that both schedules were tolerable (no dose-limiting toxicity (DLT) in the concurrent group and two grade 4 pneumonitis cases in the sequential group) and yielded a pooled one-year intracranial PFS of 35.4%, suggesting flexibility in timing without compromising early central nervous system control [26]. Focusing specifically on active brain metastases, Atlan et al. combined stereotactic radiosurgery with the same dual checkpoint regimen and reported only a single intracranial dose-limiting toxicity, an overall grade ≥3 adverse event rate of 31%, a four-month intracranial PFS of 71%, and an intracranial objective response rate of 38% after 23 months of follow-up, thereby affirming the feasibility of concurrent radio-immunotherapy in the cerebral compartment [27]. Finally, the randomized phase II PEMBRO-RT trial by Theelen et al. provided the first controlled evidence that focal SBRT can potentiate PD-1 blockade: SBRT priming almost doubled the systemic objective response rate (36% vs. 18%), prolonged mPFS from 1.7 to 6.4 months (hazard ratio [HR], 0.55), and extended median OS from 7.6 to 15.9 months (hazard ratio, 0.58) without increasing grade ≥ 3 irAEs (8% vs. 6%) [28]. Taken together, these trials consistently demonstrate that definitive LAT can be safely integrated with ICI treatment and is associated with clinically meaningful improvements in tumor response, PFS, and OS compared with outcomes expected from immunotherapy alone.

## 4. Targeted Therapy Combined with LAT in Oncogene-Addicted Disease

Randomized evidence indicates that ablative irradiation can meaningfully extend the clinical benefit of first-line TKI in EGFR-mutated tumors. The open-label, multi-institutional SINDAS trial enrolled 133 treatment-naïve patients with synchronous oligometastatic NSCLC (≤5 lesions, ≤2 per organ, no brain involvement) and an ECOG performance status of ≤2. All participants received a first-generation TKI (gefitinib, erlotinib, or icotinib); those assigned to the experimental arm also underwent stereotactic body radiotherapy to every metastatic focus and to the thoracic primary/mediastinum (25–40 Gy in five fractions, with the dose adapted to size and location). After a median follow-up of 23.6 months, upfront SBRT prolonged median PFS from 12.5 to 20.2 months (hazard ratio [HR], 0.62) and median OS from 17.4 to 25.5 months (HR, 0.68) without increasing grade 5 toxicity; symptomatic grade 3–4 pneumonitis occurred in 6% of irradiated patients versus 3% with TKI alone [29].

A contemporaneous phase III study by Sun et al. generalized this concept to third-generation therapies. In that multicenter trial, 118 patients receiving osimertinib were randomized to either continue TKI alone or receive concurrent thoracic radiotherapy (60 Gy in conventional fractions) to the primary lung tumor and involved regional nodes, with elective radiation to extra-thoracic deposits left to the investigator’s discretion. Consolidative thoracic RT significantly improved outcomes: median PFS increased from 10.6 to 17.1 months (HR, 0.57) and median OS from 26.2 to 34.4 months (HR 0.62), while three-year OS rose from 36% to 56%. Treatment-related grade ≥ 3 adverse events were higher with the combined approach (11.9% vs. 5.1%) but remained clinically acceptable, and there were no grade 5 events [30]. Together, these two phase III trials demonstrate that aggressive local ablation—delivered either upfront to all macroscopic disease or focally to the thorax—can delay resistance and translate into tangible survival gains when paired with first- or third-generation EGFR-TKIs, thereby establishing a rationale for incorporating LAT into frontline targeted therapy strategies for oncogene-addicted NSCLC.

## 5. Safety and Tolerability

All seven prospective trials included in this narrative review provided granular reporting of treatment-related toxicity, allowing a nuanced appraisal of the risk profile associated with coupling LAT with contemporary systemic regimens. In the phase II study by Bauml et al., pembrolizumab administered after definitive LAT was associated with grade 3–4 immune-related adverse events (irAE) in 12% of patients, principally pneumonitis. All events were resolved, and no treatment-related deaths were reported [24]. In contrast, the phase Ib expansion cohort reported by Bassetti et al., which combined SBRT with concurrent durvalumab plus tremelimumab, experienced a markedly higher incidence of grade ≥ 3 toxicity (40%). Only one patient had a possible relationship between the addition of SBRT and immunotherapy [25]. A randomized phase I study conducted by Bestvina et al. compared concurrent versus sequential nivolumab plus ipilimumab in conjunction with multisite SBRT. Both schedules were tolerable, with similar grade ≥ 3 adverse events associated with ICI or SBRT [26]. Focusing on intracranial disease, Atlan et al. combined stereotactic radiosurgery with dual checkpoint inhibition and documented only one intracranial dose-limiting toxicity (grade 3 radionecrosis). Overall, grade ≥ 3 treatment-related events occurred in 62% of participants, chiefly cerebral edema and pneumonitis. Two patients experienced grade 5 adverse events [27]. Reassuring safety data were also obtained in the randomized phase II PEMBRO-RT trial, where the addition of SBRT priming to pembrolizumab did not raise any new safety concerns despite significant gains in efficacy [28].

Turning to oncogene-addicted disease, the phase III SINDAS trial revealed only a modest excess of severe pneumonitis when SBRT was added to first-generation EGFR-TKIs. Grade 3–4 pulmonary toxicity occurred in 7.4% of patients in the TKI plus SBRT group versus 3% in the TKI monotherapy group [29]. Finally, a multicenter phase III study by Sun et al. demonstrated that consolidative thoracic radiotherapy delivered during osimertinib treatment increased the rate of grade ≥ 3 adverse events to 11.9% compared with 5.1% for osimertinib alone [30].

Pneumonitis was the most clinically relevant adverse event across the included trials. In the TKI setting, symptomatic grade 3–4 pneumonitis occurred in approximately 6–7% of patients receiving thoracic RT with first-generation EGFR-TKIs in SINDAS, and in 5.1% of those treated with concurrent thoracic RT plus osimertinib in Sun’s phase III trial (any-grade incidence, 32.2% vs. 1.7% with osimertinib alone) [29,30]. In the ICI setting, Bauml et al. reported pneumonitis in 11% of patients (≥G3, 6.7%) following LAT plus pembrolizumab, while the addition of SBRT in the PEMBRO-RT increased pneumonitis incidence to 26% (vs. 8% with pembrolizumab alone), with grade ≥ 3 in 11% of patients [24]. Dual ICI strategies were associated with similar risks: in COSINR, two cases of grade 4 pneumonitis (10.5%) occurred in the sequential SBRT arm [26]. Bassetti et al. observed one case of grade 3 pneumonitis (6.7%) with durvalumab/tremelimumab plus SBRT [25]. Even without thoracic irradiation, Altan et al. reported one case of grade 4 pneumonitis (8%) with nivolumab/ipilimumab plus SRS for brain metastases [27]. No treatment-related deaths (grade 5 pneumonitis) were observed.

## 6. Discussion

Although every modern study coupling LAT with ICI for omNSCLC reports a discernible incremental benefit over systemic therapy alone, the magnitude of that benefit oscillates widely across trials. Crucially, none of the ICI-containing investigations has yet advanced to a randomized phase III design. Three intertwined domains appear to drive the observed heterogeneity.

1. Biological heterogeneity at the patient level.

PD-L1 tumor proportion scores range from 0% to >90%, dictating both the probability and durability of cytotoxic T-cell engagement, while baseline tumor mutational burden, HLA diversity, neoantigen clonality, and the density of suppressive myeloid subsets further calibrate intrinsic immunogenicity.

2. Procedural heterogeneity at the trial level.

LAT can be delivered as stereotactic body radiotherapy, thermal ablation, or surgery, each with variable dose-fractionation schedules, lesion numbers, and sequencing relative to systemic therapy. The systemic backbone also differs, PD-1/PD-L1 monotherapy versus chemo-immunotherapy, and the interval between LAT completion and systemic resumption varies, all of which modulate potential synergy.

3. Methodological heterogeneity in the outcome assessment.

Definitions of the oligometastatic state, radiographic progression criteria, and primary endpoints (overall survival, progression-free survival, or time to polymitotic conversion) diverge markedly. Limited statistical power, short follow-up, and disparate censoring rules in predominantly phase I/II or single-arm phase II trials can exaggerate or obscure true biological differences. Rigorous harmonization of biomarker-based stratification, LAT delivery protocols, and endpoint frameworks, as well as progression to adequately powered phase III randomized trials, is needed to delineate the genuine additive value of LAT in this setting. The included trials used heterogeneous efficacy endpoints, including PFS, OS, oligoprogression-free survival, and time to LAT, which limited direct cross-trial comparability. To mitigate this heterogeneity, future studies should adopt a unified framework for outcome reporting. At a minimum, we recommend the consistent reporting of (i) RECIST-defined PFS, (ii) OS, and (iii) treatment-related toxicity, supplemented by site-specific local control rates for lesions treated with LAT. Harmonized definitions of oligoprogression (e.g., ≤5 new/progressive lesions on imaging) and standardized assessment intervals would enhance comparability. While exploratory or trial-specific endpoints remain valuable, the adoption of these core outcomes would facilitate meta-analyses and support the development of evidence-based guidelines. Ongoing phase III trials evaluating the incremental benefit of LAT in omNSCLC, each incorporating an ICI or TKI, are summarized in Table 3.

Variations in the sequencing and temporal spacing of LAT and ICI are equally influential in explaining the disparate efficacy signals observed in clinical trials. Front-loaded approaches, such as the PEMBRO-RT study, administer high-dose stereotactic body radiotherapy first to liberate neoantigens and trigger type I interferon signaling, thereby “priming” systemic immunity before PD-1 blockade is initiated [28]. Reverse-sequencing strategies, as exemplified by Bauml et al., initiate pembrolizumab upfront and reserve LAT for consolidating residual lesions, with the dual aim of sustaining systemic tumor control and limiting cumulative toxicity [24]. The immunological milieu of the tumor microenvironment, now that radiation is delivered—whether “cold” or already primed—dictates both the amplitude of the initial antitumor response and the selective pressures that foster resistant clones. Recent reviews have systematically highlighted how immunosuppressive components of the tumor microenvironment (TME), including myeloid-derived suppressor cells, regulatory T cells, cancer-associated fibroblasts, and inhibitory cytokine networks, undermine the efficacy of checkpoint inhibitors in NSCLC [31]. Moreover, the interval between radiation and ICI modulates lymphocyte viability, cytokine release kinetics, and tissue repair dynamics, thereby influencing efficacy and toxicity in tandem. Consequently, divergent outcomes are likely to arise from the complex interplay between patient-specific tumor biology and modality sequencing. Therefore, future investigations must adopt biomarker-guided, mechanism-driven designs to optimize the orchestration of local and systemic treatment components and achieve durable clinical benefits. Future studies should address this critical variable, the standardization of patient characteristics, treatment timing, and strategic use of combination versus single-agent therapy through larger randomized controlled trials. This approach will better delineate the patient populations that are most likely to benefit from specific ICI strategies combined with LAT.

Collective evidence indicates that the addition of LAT to modern systemic regimens is promising for de novo omNSCLC. Phase II trials showed meaningful gains in PFS and OS. However, definitive proof remains pending: large phase III trials, such as ANDROMEDA (chemo-immunotherapy ± LAT, NCT06141070) and NIRVANA-LUNG (pembrolizumab ± SBRT, NCT03774732), are currently recruiting, and their results will determine whether LAT becomes routine in first-line care. Ongoing phase III trials investigating the incremental benefits of LAT in omNSCLC are summarized in Table 3. Although genomic aberrations beyond EGFR—most notably rearrangements in ALK, ROS1, and RET— have been cataloged, the extant dataset is too scant to permit a statistically robust interrogation. Their low prevalence, coupled with the paucity of prospective cohorts, effectively precludes the assembly of adequately powered phase III trials. It is biologically plausible that the survival advantage conferred by combining LAT with tyrosine kinase inhibition in EGFR-mutant disease might extrapolate to these less common molecular subtypes. The biological rationale for extending the LAT-plus-TKI paradigm beyond EGFR to other oncogene-addicted driver genes is grounded in the shared principle of oncogene addiction. In these subsets, tumor growth remains highly dependent on the driver kinase pathway, and resistance often emerges as spatially restricted clonal events rather than as systemic escape. This pattern of oligoprogression suggests that ablating resistant lesions while maintaining effective systemic inhibition can prolong disease control [32]. Therefore, preclinical models are indispensable for substantiating and mechanistically dissecting biological plausibility. Patient-derived xenograft (PDX) models provide a powerful translational tool for exploring the mechanisms of resistance and oligoprogression under systemic therapy. Because PDX tumors retain the genomic and histopathological features of the original patient tumor, they can recapitulate clonal evolution during TKI treatment and help rationalize the need for local ablative therapy to eradicate focal resistant clones. [33].

Regarding safety, current evidence demonstrates that LAT can be combined with ICI or EGFR-TKI without yielding unanticipated adverse signals. Collectively, these data indicate that, although dual checkpoint blockade paired with multisite high-dose radiation and wide-field thoracic irradiation during TKI therapy increases the incidence of grade ≥ 3 toxicity to approximately 25–40%, elevated irAE rates with dual ICI are also seen in trials that do not include LAT and are comparable to those reported in other CTLA-4-based combination studies [9,10,11]. No novel safety concerns were observed. Pneumonitis and other pulmonary complications represent the most consistent risks, particularly in patients with prior thoracic irradiation or extensive target volumes. Consequently, rigorous baseline pulmonary evaluation, vigilant radiographic monitoring, and prompt initiation of corticosteroid therapy are essential to mitigate treatment-related lung injury. When these precautions are in place, the clinical benefits of LAT appear to outweigh its incremental toxicity in appropriately selected patients.

## 7. Future Perspectives

Selecting the right patients is the key to making LAT truly useful for oligometastatic NSCLC. Future studies should use several types of tests, including standard scans, metabolic imaging, and blood-based markers, to determine the extent of residual disease after first-line systemic treatment.

Imaging tools. A post-induction 18F-FDG PET/CT scan remains one of the most useful methods. When the scan shows persistent “hot” spots, it usually means that live tumor cells are still present; therefore, adding LAT can aim to remove or destroy those areas and improve control [34]. In contrast, a complete metabolic response (no hot spots) suggests that only tiny amounts of cancer remain; in this setting, extra local treatment may provide little extra benefit and could increase side effects.

Molecular tools. Changes in circulating tumor DNA (ctDNA) offer another simple way to follow the disease. Several reports show that when ctDNA disappears after systemic therapy, long-term disease control is much more likely, even if small lesions can still be seen on scans [35]. From a pragmatic standpoint, the integration of LAT should be considered in patients with limited tumor burden (generally ≤5 lesions), durable disease control on systemic therapy, and favorable performance status. The choice of local modality should be guided by disease site—SRS/SRT for brain, SBRT or surgery for lung and adrenal, and SBRT for bone lesions—balancing efficacy with toxicity and institutional expertise. While these criteria remain provisional and are largely drawn from early-phase trials, they provide a pragmatic framework to guide clinical decision-making pending phase III validation.

New research directions. Radiomics and machine-learning approaches show promise for predicting progression and treatment benefit in NSCLC. For example, Khorrami et al. demonstrated that intra- and peritumoral CT radiomic features correlate with immunotherapy response and overall survival, suggesting that image-derived phenotypes may capture immune-relevant biology with prognostic value [36]. Nonetheless, current evidence is limited by retrospective designs, multi-scanner and segmentation variability, and incomplete external validation; thus, prospective, multi-center studies with standardized pipelines will be required to establish generalizability and clinical utility. Moreover, selection based on the number (e.g., ≤3 vs. 4–5 lesions) and anatomical distribution of metastases may further refine the eligibility for local consolidation.

## 8. Conclusions

In summary, LAT combined with ICIs or TKIs is emerging as a promising strategy for omNSCLC. However, unresolved issues—optimal timing, lesion scope, toxicity management, and biomarker-guided selection—must be clarified through ongoing phase III trials and biomarker-driven protocols before the approach can be considered the standard of care. Future research should prioritize adequately powered, multicenter randomized phase III trials that adopt harmonized endpoints such as OS, RECIST-defined PFS, and site-specific local control. Patient selection should be prospectively stratified by tumor burden (≤5 lesions), disease distribution (brain, adrenal, and lung), and biomarker context, including ctDNA clearance and PD-L1 expression. Toxicity reporting must be standardized (CTCAE v5) with mandated pulmonary function testing and early imaging to ensure comparability. In parallel, translational efforts leveraging patient-derived xenograft models, organoids, and radiomics/ctDNA integration are warranted to dissect mechanisms of oligoprogression and resistance. Collectively, these steps will provide the methodological rigor necessary to translate current promising signals into evidence-based guidelines for omNSCLC.

## Figures and Tables

**Table 1:** Characteristics of included studies.

Lead Author/Year (Trial)	Design	Number of Metastases	Systemic Therapy	LAT	Primary Endpoint
Bauml 2019 [24]	Phase II, single-arm	≤5 lesions/≤3 organs	Pembrolizumab	Surgery or SBRT	2-yr PFS rate
Bassetti 2023 [25]	Phase Ib, dose-expansion	≤5 lesions	Tremelimumab + Durvalumab	SBRT	Grade ≥ 3 treatment-related AEs
Bestvina 2022 [26]	Randomised Phase I	No limit	Nivolumab + Ipilimumab	SBRT	Dose-limiting-toxicity rate
Altan 2023 [27]	Phase I/II	Not mentioned	Nivolumab + Ipilimumab	SRS to brain metastases	Intracranial PFS
Theelen 2019 (PEMBRO-RT) [28]	Randomised Phase II	No limit (1 lesion irradiated)	Pembrolizumab	SBRT	12-wk ORR
Wang/Bai 2021 (SINDAS) [29]	Phase III	≤5 lesions/≤2 organs	Gefitinib/Erlotinib	SBRT	PFS
Sun 2024 [30]	Phase III	≤5 lesions/≤2 organs	Osimertinib	Thoracic RT	PFS

AE, adverse event; ORR, objective response rate; LAT, local ablative therapy; PFS, progression-free survival; SBRT, stereotactic body radiotherapy; RT, radiotherapy.

**Table 2:** Main results of included studies.

Lead Author/Year (Trial)	Study Design	PFS	OS	Grade ≥ 3 Toxicity
Bauml 2019 [24]	Pembrolizumab consolidation after complete LAT	mPFS; 19.1 mo	2-yr OS 77.5%	12.2%
Bassetti 2023 [25]	Tremelimumab + Durvalumab with multisite SBRT	mPFS; 42 mo	NR	40%
Bestvina 2022 [26]	Concurrent vs. sequential Nivolumab + Ipilimumab + multisite SBRT	mPFS; 5.8 mo (concurrent)/4.7 mo (sequential)	NR	73%
Altan 2023 [27]	Brain SRS + Nivolumab/Ipilimumab	1-yr intracranial PFS; 35.4%	NR	8%
Theelen 2019 (PEMBRO-RT) [28]	Pembrolizumab + single-site SBRT vs. Pembrolizumab	mPFS; 6.6 mo vs. 1.9 mo	mOS; 15.9 mo vs. 7.6 mo	46%
Wang/Bai 2021 (SINDAS) [29]	1st generation TKI ± up-front SBRT	mPFS; 20.2 mo vs. 12.5 mo	mOS; 25.5 mo vs. 17.4 mo	7.4% (pneumonitis)
Sun 2024 [30]	Osimertinib + concurrent thoracic RT vs. Osimertinib	mPFS; 17.1 mo vs. 10.6 mo	mOS; 34.4 mo vs. 26.2 mo	11.9% vs. 5.1%

NR, not reached; LAT, local ablative therapy; OS, overall survival; PFS, progression-free survival; RT, radiotherapy; SBRT, stereotactic body radiotherapy; TKI, tyrosine kinase inhibitor.

**Table 3:** Ongoing/recruiting phase III trials evaluating local ablative strategies combined with immune checkpoint inhibitors or tyrosine kinase inhibitors in oligometastatic stage IV NSCLC.

NCT Number (Trial)	Systemic Treatment Regimen	Timing of LAT	Maximum No. of Metastatic Lesions	Primary Endpoint
NCT03391869 (LONESTAR)	Nivolumab + Ipilimumab	After two induction ICI cycles (≈12 weeks): randomized to continue ICI ± definitive surgery or high-dose RT to all sites	≤3 lesions	OS
NCT03827577 (OMEGA)	Biomarker-driven standard therapy: platinum doublet, EGFR-TKI, or ICI	If progression-free after ~3 months, randomized to continue systemic therapy ± consolidative LAT (surgery, SBRT, RFA)	≤3 lesions	OS
NCT03774732 (NIRVANA-Lung)	Pembrolizumab + platinum-based chemotherapy	Multisite hypofractionated RT delivered early (cycle 2) vs. none	≤5 lesions	1-year OS
NCT05278052 (TARGET-02)	Standard maintenance therapy (e.g., pemetrexed ± pembrolizumab) after induction chemo(±ICI)	During maintenance phase: systemic therapy ± SBRT to every residual lesion	≤5 lesions (≤3 per organ)	OS
NCT06141070 (ANDROMEDA)	Chemo-immunotherapy or ICI alone	Up-front high-dose SBRT to all lesions within first two systemic cycles vs. none	≤5 lesions	OS and PFS

EGFR, epidermal growth factor receptor; ICI, immune-checkpoint inhibitors; LAT, local ablative therapy; OS, overall survival; PFS, progression-free survival; RT, Radiotherapy. SBRT, Stereotactic body radiotherapy; TKI, Tyrosine kinase inhibitor.
